# *Porphyromonas gingivalis*, a bridge between oral health and immune evasion in gastric cancer

**DOI:** 10.3389/fonc.2024.1403089

**Published:** 2024-05-14

**Authors:** Matías Muñoz-Medel, Mauricio P. Pinto, Lauren Goralsky, Mónica Cáceres, Franz Villarroel-Espíndola, Patricio Manque, Andrés Pinto, Benjamin Garcia-Bloj, Tomas de Mayo, Juan A. Godoy, Marcelo Garrido, Ignacio N. Retamal

**Affiliations:** ^1^Precision Oncology Center, School of Medicine, Faculty of Medicine and Health Sciences, Universidad Mayor, Santiago, Chile; ^2^Support Team for Oncological Research and Medicine (STORM), Santiago, Chile; ^3^Department of Biological Sciences, Columbia University, New York, NY, United States; ^4^Millennium Nucleus of Ion Channel-Associated Diseases (MiNICAD), Santiago, Chile; ^5^Program of Cellular and Molecular Biology, Institute of Biomedical Sciences (ICBM), Faculty of Medicine, Universidad de Chile, Santiago, Chile; ^6^Millennium Institute on Immunology and Immunotherapy, Faculty of Medicine, Universidad de Chile, Santiago, Chile; ^7^Translational Medicine Laboratory, Fundación Arturo López Pérez Cancer Center, Santiago, Chile; ^8^Department of Oral and Maxillofacial Medicine and Diagnostic Sciences, Case Western Reserve University School of Dental Medicine, Cleveland, OH, United States

**Keywords:** *P. gingivalis*, microbiome, PAMPS, gastric cancer, PD1/PDL1 axis

## Abstract

*Porphyromonas gingivalis* (*P. gingivalis*) is a gram-negative oral pathogen associated with chronic periodontitis. Previous studies have linked poor oral health and periodontitis with oral cancer. Severe cases of periodontal disease can result in advanced periodontitis, leading to tissue degradation, tooth loss, and may also correlate with higher gastric cancer (GC) risk. In fact, tooth loss is associated with an elevated risk of cancer. However, the clinical evidence for this association remains inconclusive. Periodontitis is also characterized by chronic inflammation and upregulation of members of the Programmed Death 1/PD1 Ligand 1 (PD1/PDL1) axis that leads to an immunosuppressive state. Given that chronic inflammation and immunosuppression are conditions that facilitate cancer progression and carcinogenesis, we hypothesize that oral *P. gingivalis* and/or its virulence factors serve as a mechanistic link between oral health and gastric carcinogenesis/GC progression. We also discuss the potential impact of *P. gingivalis*’ virulence factors (gingipains, lipopolysaccharide (LPS), and fimbriae) on inflammation and the response to immune checkpoint inhibitors in GC which are part of the current standard of care for advanced stage patients.

## 1 Introduction

In recent decades, significant advances in sequencing technologies have expanded our knowledge on the microbiome, sparking researchers’ interest in the role of the human microbiome in homeostasis and disease. Consensus establishes that many diseases, ranging from Alzheimer’s to endocrine disorders, and cancer, are associated with microbial imbalances or dysbiosis (1).

The oral cavity is the entry portal to the gastrointestinal tract. Changes in the oral cavity’s bacterial diversity can have local and systemic consequences on health and disease (2, 3). Within the oral cavity, bacteria can be found in the saliva or as a part of biofilm-structured communities. Interestingly, previous studies have linked poor oral health and chronic periodontitis with oral cancer (4). Severe cases of periodontitis can result in tissue degradation and tooth loss, potentially correlating with an elevated risk of developing gastric cancer (GC). However, the existing evidence from case-control studies and five cohort studies regarding tooth loss as a potential marker remains inconclusive. Significant heterogeneity among studies and mixed results between case-control and cohort studies contribute to this association’s uncertainty (5).

## 2 Is *Porphyromonas gingivalis* the link between the oral microbiome and gastric cancer?

Perhaps the best example of an association between dysbiosis and cancer is *Helicobacter pylori* (*H. pylori*) and GC. This gram-negative bacterium colonizes the stomach and plays a central role in developing this pathology. *H. pylori* is widely recognized as an oncogenic factor and a driver of gastric carcinogenesis. In fact, the International Agency for Research on Cancer (IARC) classifies the *H. pylori* infection within the group I of human carcinogens (6). Within the gastric mucosa, *H. pylori* modulates acid secretion, affecting the gastric microbiome, leading to further dysbiosis, *H. pylori* overgrowth, and associated diseases. Current hypotheses suggest that recurrent and persistent *H. pylori* infections cause chronic inflammation, eventually leading to gastric carcinogenesis (7). In addition to *H. pylori*, new evidence suggests that other bacteria, including oral bacteria and their metabolites, may contribute to gastric carcinogenesis and GC progression (8, 9).

The oral microbiome is the most complex and dynamic arrangement of microbial communities within the human body. Changes in the oral microbiome can have profound consequences in an individual’s homeostasis. As pointed out earlier, periodontitis is a chronic inflammatory disease characterized by oral dysbiosis. Recent studies postulate that the transition from a healthy state into dysbiosis is driven by specific “keystone pathogens” that alter the host immune system, modifying the conditions of the microenvironment and disrupting the balance among bacterial communities.

*Porphyromonas gingivalis* (*P. gingivalis* or *Pg*) is a gram-negative, anaerobic, rod-shaped oral bacteria, and a keystone pathogen in chronic periodontitis (10). Despite its role in periodontitis, high levels of *P. gingivalis* have also been documented in healthy subjects without oral disease (11). Unlike other oral bacteria, *P. gingivalis* is an acid-resistant bacterium and studies demonstrate it is able to migrate from the oral cavity to the colon (12, 13). This suggests that *P. gingivalis* in the ingested saliva may easily reach the stomach. In addition to being swallowed, everyday activities such as brushing, flossing, chewing, and dental procedures cause transient *P. gingivalis* bacteremia (14, 15). Some of these activities can result in the translocation of the bacterium into other tissues, such as the liver, placenta, or even coronary arteries (16). Similar to other bacteria, *P. gingivalis* possess a variety of virulence factors, including gingipains (cysteine proteases), capsule, lipopolysaccharides (hereafter called *Pg*-LPS), and fimbriae. These factors modulate systemic inflammation, dysbiosis, tumorigenesis, and contribute to the evasion of the innate immune response. In one hand, gingipains are responsible for the development of periodontitis (16, 17), inducing cell migration and the release of other pro-inflammatory mediators (18). On the other hand, *Pg-*LPS is responsible for the maintenance of chronic inflammation by increasing the secretion of proinflammatory cytokines. Interestingly, a recent article reports that *P. gingivalis* and its gingipains may play a central role in other chronic, age-related pathologies, such as Alzheimer’s disease (19). Also, studies in rats demonstrate that topical applications of Pg-LPS lead to severe complications, including neuroinflammation and impaired learning and memory (20).

### 2.1 The contribution of *P. gingivalis* in carcinogenesis and cancer progression

**Figure 1:**
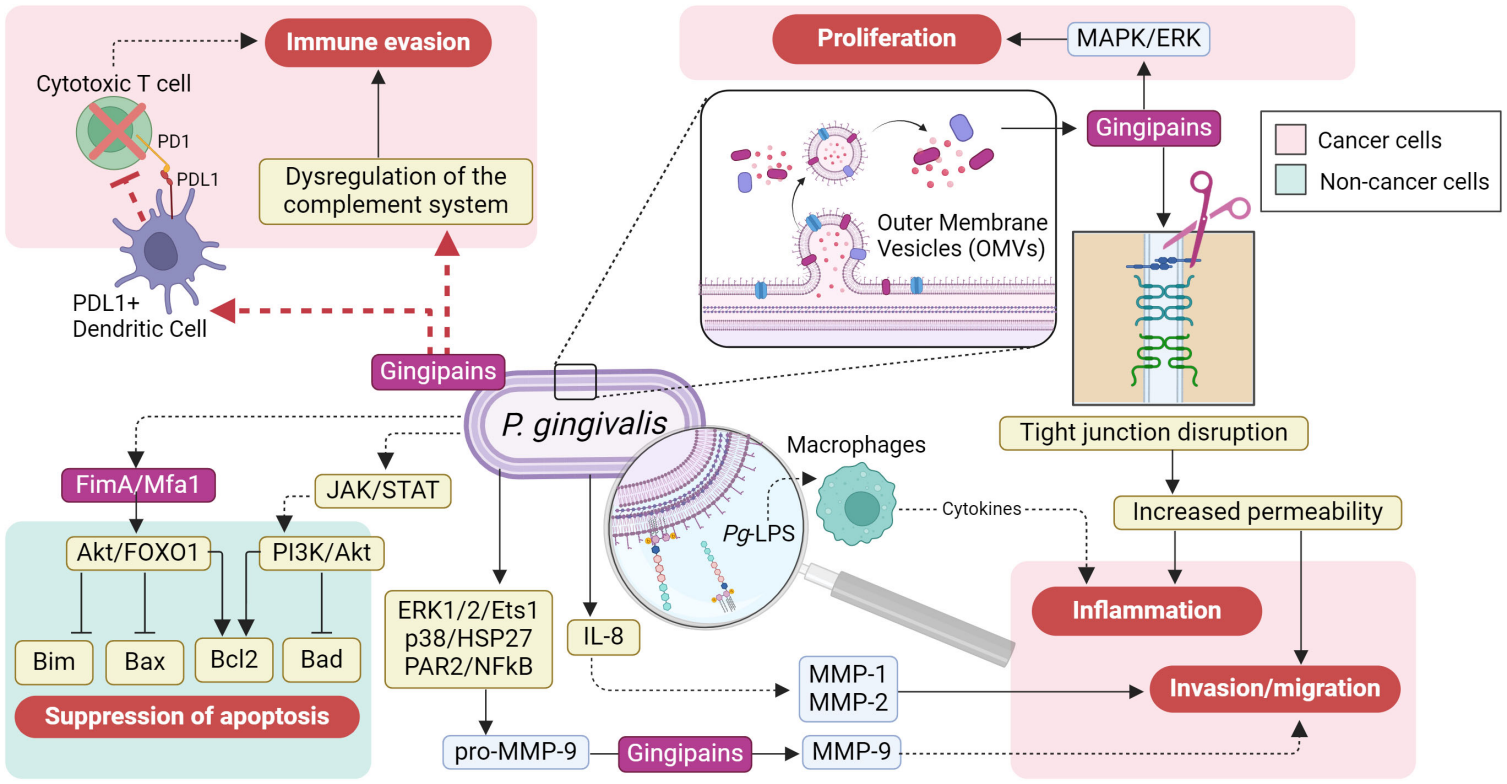
The cancer-promoting effects of *Porphyromonas gingivalis* on cancer cells. The cancer-promoting effects on gastric epithelial cells are mediated through its virulence factors, which include pathogen-associated molecular patterns (PAMPs) like lipopolysaccharides (LPS), gingipains, and fimbriae. These factors can directly trigger immune evasion, inflammation, invasion/migration, and proliferation by activating numerous molecular pathways in cancer cells (depicted in the light red boxes); these pathways are potentially influenced by the PD1/PDL1 axis (shown by the red dashed arrow). Additionally, *P. gingivalis* virulence factors may contribute to the suppression of apoptosis by activating signaling pathways, such as JAK/STAT, PI3K/AKT, and AKT/FOXO1, thereby promoting the progression of gastric cancer (shown in the light green box) (Figure created in BioRender.com).

Studies demonstrate that serum antibodies against *P. gingivalis* are associated with orodigestive cancer mortality (21) suggesting a role in cancer risk. Previous studies have reported the effects of *P. gingivalis* and its virulence factors upon some of the “hallmarks of cancer” (22). As summarized in **Figure 1**, *P. gingivalis* can mediate immune evasion and increase inflammation in cancer cells; it also stimulates proliferation and invasion/migration. Reports in gingival epithelial cells indicate that *P. gingivalis* also suppresses apoptosis.

As proteases, *Pg*-gingipains can degrade components of the tight junctions within the gingival and the gastrointestinal epithelia, disrupting their integrity (23, 24). A coordinated attack of *Pg*-gingipains and *H. pylori* toxins can severely damage the gastric epithelium, promoting an aggravated state of vulnerability with increased permeability to pathogens and virulence factors, leading to reduced functionality, chronic gastritis, and systemic inflammation (25–27). In GC patients, the degradation of tight junctions also facilitates the spread of cancer cells throughout the body, promoting metastasis (26, 28, 29).

Another mechanism by which *Pg*-gingipains can increase GC progression is by cleavage, phosphorylation, and degradation of the 27-kDa heat shock protein (HSP27), a chaperone that responds to stress and apoptosis (30–34). Then, HSP27 reduction impairs cells’ ability to respond to stress signals and their protection against damage, thereby contributing to GC progression.

Additionally, *P. gingivalis* can disrupt apoptosis by upregulating B-cell lymphoma proteins (Bcl2), a family of direct and indirect proapoptotic proteins, via virulence factors such as fimbriae, gingipains, and hemaphore-like proteins. This phenomenon has been observed in *P. gingivalis*-infected dendritic and CD3+ T cells as a mechanism to prolong the survival of infected cells in parallel to the host immunity evasion (35).

Similarly, *P. gingivalis* Mfa1 and FimA fimbriae also exhibit antiapoptotic activity by upregulating C-X-C chemokine receptor type 4 (CXCR4) and downregulating Forkhead Box O1/3 (FOXO1/3), which are associated with tumor progression (36). Moreover, *Porphyromonas* sp. can confer cancer cells with enhanced tumor invasion and metastatic capabilities by promoting matrix metalloproteinase 9 (MMP9), an extracellular matrix degrader, through activation of protease-activated receptor 2 (PAR2) and the ERK1/2-Ets1/3-p38 pathway (37, 38) (**Figure 1**).

Provided that the oral cavity is the entry portal to the gastrointestinal tract, *P. gingivalis* and its virulence factors have all been implicated in the carcinogenesis and progression of oral, esophageal, liver, colorectal, and pancreatic cancers. The fact that distant organs can be affected emphasizes that *P. gingivalis* has systemic tumorigenic and tumor enhancer effects (39–41). The damage mechanisms of *P. gingivalis* may include biological processes such as modifying and evading the innate immune response, promoting inflammation, and suppressing apoptosis (42, 43) (**Figure 1**).

### 2.2 How does *Porphyromonas gingivalis* evade the immunological surveillance?

*P. gingivalis* is a well-established microbial modulator that drives dysbiosis by activating Toll-like receptors (TLRs), resulting in a shift in the immune response in favor of the pathogens (44). In this context, *Pg*-LPS exhibits low endotoxin activity and structural variation, hence, the differential activation of TLR2 and TLR where *Pg*-LPS is predominantly recognized by TLR2 (45). In addition, another virulence factor of *P. gingivalis*, the fimbriae, stimulates cytokine expression by interacting with TLRs. The host cells recognizes the fimbriae as a potential threat to the immune system, and their detection via Pattern Recognition Receptors (PRRs) regulates the induction of immune costimulatory molecules that facilitate the initiation of T cell-mediated immunity. However, in persistent *P. gingivalis* infections, fimbriae can upregulate the expression of costimulatory molecules, thereby exacerbating inflammation mechanisms by activating the response mediated by CD4+ T cells (46).

Furthermore, gingipain degrades proinflammatory cytokines such as interleukin (IL)-1β, tumor necrosis factor (TNF)-α, interferon (IFN)-γ, IL-12, IL-8, IL-6, and their receptors, altering host defense mechanisms associated with inflammation. It also activates matrix metalloproteinases (MMPs), such as MMP-1, MMP-3, and MMP-9, conferring an aggressive, invasive, and prometastatic state to tumor cells (47, 48).

In summary*, P. gingivalis* may enhance tumor progression to an advanced stage from oral disease by translocating, enriching the tumor microenvironment with virulence factors, manipulating the immune response, maintaining chronic inflammation, and stimulating changes in the host’s immunological surveillance that ultimately serve to promote GC and facilitate the systemic progression of this disease.

## 3 Hypothesis: oral *Porphyromonas gingivalis* as a driver of gastric cancer progression

We hypothesize that oral *P. gingivalis* infection contributes to GC progression by increasing *Pg*-LPS, PAMPs, *Pg*-gingipains, inflammatory mediators, and immune checkpoint components, such as the PD1/PDL1 axis. Our hypothesis was based on the fact that most oral pathogens are unlikely to survive in an acidic stomach environment. Additionally, it has been observed that *P. gingivalis*, when present in oral plaque, is associated with a higher risk of precancerous gastric lesions (49). Furthermore, individuals with periodontal disease and high levels of colonization of periodontal pathogens are related to high levels of immunoglobulin G (IgG) anti-*P. gingivalis* in the serum of patients, indicating that this pathogen may also increase the mortality risk of cancer patients through other independent mechanisms associated with periodontitis (21).

A plausible explanation for this association could involve a “*hit-and-run*” mechanism in which transiently resident *P. gingivalis* is rapidly inactivated and destroyed by the acidic stomach environment, avoiding the installment of a local infection. However, bacteria can still release virulence factors such as *Pg*-LPS and gingipain-containing outer membrane vesicles (OMVs) locally and systemically (50) (**Figure 1**).

**Figure 2:**
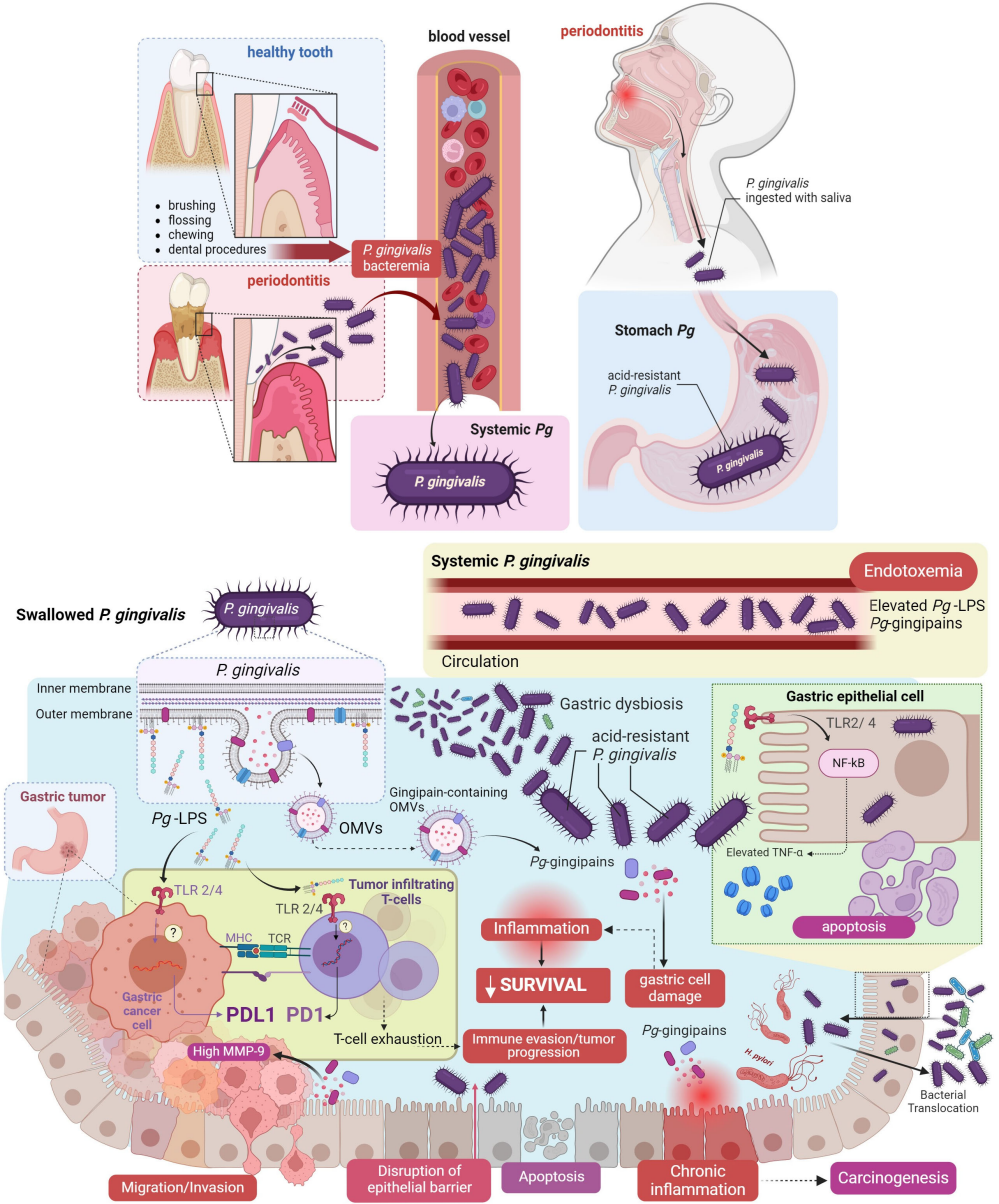
Hypothesis: *Porphyromonas gingivalis* and its pathogenic factors in oral health enhance immune evasion in gastric cancer. Oral infection with *P. gingivalis* can lead to bacterial dissemination through the bloodstream or via swallowing of saliva, reaching the stomach (depicted in the light blue box). *P. gingivalis*, utilizing its virulence factors, may induce gastric dysbiosis upon reaching the gastric epithelial cells, triggering inflammation, and reducing cell survival. These processes occur between tumor cells and the immune system and facilitate the infiltration of T-cells into the tumor, T-cell exhaustion, and activation of the PD1/PDL1 axis enhance immune evasion (illustrated in the yellow box). All these processes constitute a significant part of the mechanism proposed in our hypothesis. These cellular events progress from migration/invasion, disruption of the epithelial barrier, apoptosis, to chronic inflammation, ultimately culminating in carcinogenesis. (Figure created in BioRender.com).

According to our hypothetical model, poor oral health generates a bacterial imbalance, or dysbiosis, that reduces the diversity of local bacteria (**Figure 2**). Thus*, P. gingivalis* becomes dominant in a process that can lead to severe chronic periodontitis and tissue destruction (51). Uncontrolled proliferation of *P. gingivalis* increases the production and delivery of virulence factors such as PAMPs, mainly *Pg*-LPS and gingipains via OMVs, impairing the host immune surveillance and further potentiating oral dysbiosis (52). At this stage, *P. gingivalis* could be translocated systemically to other organs. Previous studies have demonstrated the presence of *P. gingivalis* DNA in the cerebrospinal fluid of living subjects diagnosed with probable Alzheimer’s disease (19, 53). Subsequently, *Pg*-LPS and OMVs-containing gingipain reach the bloodstream and the lymphatic system via gingival vessels. Then, following our arguments to consolidate the hypothesis, *Pg*-LPS accesses the lymphatic ganglia and the gastric tumor microenvironment, where it binds to TLR4 receptors, increasing the expression of ligands and receptors of the immune checkpoint pathway (PD1/PDL1 axis). Activation of TLR4 receptors by *Pg*-LPS triggers the NF-κB pathway and production of pro-inflammatory cytokines in innate immune cells (54). Studies on peripheral T-cell lymphomas have shown that TLR4 overexpression is associated with higher PDL1 expression, translating into poorer prognosis (55).

Additionally, studies in mice suggest that TLR4 activation by systemic *Pg*-LPS can modulate the response to PD1 therapy during chronic viral infection (56). Therefore, following our line of argument, TLR4 activation by *Pg*-LPS increases PD1/PDL1 expression in T-cells and GC cells, leading to simultaneous T-cell exhaustion by PD1 and an increase in PDL1, which allows gastric tumor progression, culminating in a decrease in patient survival which evidences this fact (**Figure 2**).

Interestingly, studies combining colorectal cancer cell lines and mouse models have demonstrated that *Pg*-LPS promotes metastasis by increasing cell invasion and migration via NF-κB (57). Furthermore, regarding the association between PD1/PDL1 expression and cancer patients’ survival, a Japanese study showed that PDL1 expression was associated with worse overall survival in stage II/III GC patients (58). However, this association has been inconsistent among other malignancies, showing no association or improved patient survival in some cancers, and remain controversial (59–61).

## 4 Discussion

A dysbiotic oral microbiome may contribute to the development of both local (oral) and systemic diseases. This includes a wide range of illnesses from dental caries and periodontal disease (62, 63) to cardiovascular disease and cancer (64, 65). A genomic study analyzed the composition of the digestive tract microbiome and reported a 45% of overlap between oral and stool bacteria (66), suggesting the transfer of oral bacteria into the stomach is not uncommon. In fact, ingested saliva contains a great amount of oral bacteria. Although these are usually unable to colonize a healthy gut they have been reported in the intestine of individuals that suffer from colon cancer and inflammatory bowel disease, among others (67). This supports the idea of the oral cavity as a reservoir for potential gastrointestinal pathogens, which in turn can exacerbate gastrointestinal diseases.

However, microbiota components also induce immune evasion mechanisms to restore homeostasis in the host organism. Previous studies have demonstrated a correlation between various cancers, the presence of *P. gingivalis*, and its diverse virulence mechanisms. Additionally, these studies highlighted the activation of immune checkpoint mechanisms in the host.

While the prognostic significance of PDL1 in gastric carcinomas remains uncertain, certain studies have highlighted its relevance in clinical outcomes across the entire cohort via multivariate analysis. Specifically, PDL1(+) tumors were identified as an adverse prognostic factor in Epstein Barr Virus (EBV)-positive carcinomas but not MSI-high carcinomas. Also, CD8+ tumors have shown low tumor-infiltrating lymphocytes, and more advanced-stage tumors were associated with unfavorable clinical outcomes. Furthermore, the knockdown of PDL1 in gastric carcinoma cells demonstrated significant suppression of proliferation, invasion, and cell migration while increasing apoptosis. Notably, these effects were prominent in two EBV(+) cell lines but not consistently observed across all three EBV (–) cell lines (68).

An investigation on PDL1 levels in a prostate cancer cell line revealed that infection with *P. gingivalis* and its PAMPs positively influenced PDL1 expression. This positive regulation was facilitated through the nucleotide-binding oligomerization domain (NOD)1/NOD2 signaling pathway. Interestingly, no upregulation was observed following the treatment of cells with *Pg*-LPS. These findings suggest that chronic inflammatory conditions within the organ might play a role in tumor immune evasion by altering the tumor microenvironment (69).

In several studies conducted on different cancer types, such as PC, it has been observed that microbiota communities within tumors partially overlap with those found in the oral cavity. For instance, *P. gingivalis* has been found to aggregate significantly more in PC tissues than in adjacent normal tissues, thereby altering the tumor microenvironment and promoting pancreatic tumorigenesis in murine models. This altered microenvironment is characterized by a notable increase in neutrophil enrichment and a significant decrease in CD8+ cytotoxic T cells. In contrast, no significant changes were observed in CD4+ T cells and monocytes. These findings suggest that *P. gingivalis* may induce a neutrophil-dominated proinflammatory response in mice with PC, contributing to the suppression of the tumor immune environment (70).

Elevated levels of anti-*P. gingivalis* antibodies have been identified in patients with pancreatic cancer (PC) compared to healthy controls. Moreover, *P. gingivalis* and other microbiota members possess peptidyl arginine deaminase (PAD) enzymes. Together with mutations in the arginine of tumor protein p53 (TP53), these factors establish a close relationship between a microbiota component, virulence factors, and the progression of PC (71).

Current evidence supports the significant role of PDL1 in carcinogenesis, particularly in hepatocellular carcinoma (HCC), where the bidirectional regulation of PDL1 mediated by TP53/mechanistic Target of Rapamycin Complex 1 (mTORC1) has been elucidated. In HCC with non-mutated TP53, mTORC1 suppression leads to increased E2F1 transcription factor expression. This increase interrupts the cytoplasmic interaction between E2F transcription factor 1 (E2F1) and PDL1, facilitating the translocation of E2F1 into the nucleus, where it positively activates PDL1 transcription. Conversely, in HCC with mutated TP53, mTORC1 suppression promotes PDL1 protein degradation through autophagy. Additionally, it has been observed that tumor infiltration by CD8+ T cells is significantly reduced in this type of HCC. These findings highlight the pivotal role of TP53 in regulating PDL1 expression and modulating immune evasion in HCC (72).

Investigating the microbiota in low biomass environments like the stomach and lungs poses significant challenges for molecular studies, primarily due to the difficulty in detecting pathogenic agents. Nonetheless, these environments also display reduced microbial complexity, potentially limiting the number of causal agents and mitigating the risk of establishing spurious causal relationships (73, 74). Randomized control trials and prospective cohorts can confirm or refute the model that links GC, PD1/PDL1 axis, and periodontitis-related pathogens (75).

In the realm of human pathologies, the investigation into potential standard causal links between periodontitis and rheumatoid arthritis (RA) has revealed a connection between the ability of *P. gingivalis* to citrullinate peptides and the presence of autoantibodies against citrullinated peptides, which are particular and sensitive markers in the diagnosis of RA. Consequently, peptide citrullination has been implicated in triggering an autoimmune response, whereby the immune system perceives modified self-proteins and peptides as foreign. While citrullinated peptides may play a role in the pathogenesis of RA, the precise nature of their emergence and function remains unclear, and the potential contributions of the microbiota in the citrullination process are yet to be fully elucidated (76, 77).

Ultimately, prospective studies should evaluate oral health status at diagnosis and utilize next-generation sequencing (NGS) tools to fully examine the presence of specific oral bacteria and virulence factors. Enhanced methodologies for elucidating causal relationships and experimental disease models will contribute to a more comprehensive assessment of the role played by particular microbiome organisms and their virulence factors in GC onset, progression, and response to anticancer treatment (78), taking into account that the experimental design of such studies are the main cause of overstating or understating the importance of certain pathogens or microbiome profiles on cancer, as was detailed by a critical review (79).

### 4.1 An experimental approach to our hypothesis: microbiota, metabolites, and evasion of the host immune response

The experimental approach to our hypothesis encompasses three key axes. First, elucidating the relationship between *P. gingivalis*, periodontal disease, and the progression of digestive tract tumors, particularly GC, would require a large-scale prospective multicenter study employing transcriptome RNA sequencing to characterize the metabolically active microbiota. This analysis should encompass bacteria in the gastrointestinal tract and within tumors (80). The second aspect involves identifying metabolites produced by the bacterial community (metabolomics) to obtain a comprehensive overview of metabolites, including amino acids, carbohydrates, carbohydrate conjugates, fatty acids, glycerophospholipids, nucleosides, and nucleotides. This analysis and its findings would pave the way for the third aspect of our hypothesis, which aims to pinpoint key metabolites, such as adenosine, a classic regulator of metabolic and immunological checkpoints, that participate in the tumor’s evasion of the host immune system.

Finally, studies have reported increased nucleoside concentrations in GC patients with peritoneal recurrence compared with those without peritoneal recurrence. Unraveling this intricate landscape would enable us to identify the metabolites produced by *P. gingivalis* that manipulate the host’s immune system. Consequently, this phenomenon may inadvertently facilitate cancer cell progression towards tumorigenesis as a secondary effect (80, 81).

## Data availability statement

The original contributions presented in the study are included in the article/supplementary material. Further inquiries can be directed to the corresponding author.

## Author contributions

MM-M: Conceptualization, Writing – original draft, Writing – review & editing. MP: Conceptualization, Writing – original draft, Writing – review & editing, Visualization. LG: Writing – original draft, Writing – review & editing. MC: Writing – original draft, Writing – review & editing. FV-E: Writing – original draft, Writing – review & editing. PM: Writing – original draft, Writing – review & editing. AP: Writing – original draft, Writing – review & editing. BG-B: Writing – original draft, Writing – review & editing. TM: Writing – original draft, Writing – review & editing. JG: Writing – original draft, Writing – review & editing. MG: Writing – original draft, Writing – review & editing. IR: Writing – original draft, Writing – review & editing, Conceptualization, Funding acquisition, Supervision, Visualization.

## References

[1] Thomas S Izard J Walsh E Batich K Chongsathidkiet P Clarke G. The host microbiome regulates and maintains human health: A primer and perspective for non-microbiologists. Cancer Res. (2017) 77:1783–812. doi: 10.1158/0008-5472.CAN-16-2929 PMC5392374 28292977

[2] Kozak M Pawlik A. The role of the oral microbiome in the development of diseases. Int J Mol Sci. (2023) 24:5231. doi: 10.3390/ijms24065231 36982305 PMC10048844

[3] Tan X Wang Y Gong T. The interplay between oral microbiota, gut microbiota and systematic diseases. J Oral Microbiol. (2023) 15:2213112. doi: 10.1080/20002297.2023.2213112 37200866 PMC10187086

[4] Zhang L Liu Y Zheng HJ Zhang CP. The oral microbiota may have influence on oral cancer. Front Cell Infect Microbiol. (2019) 9:476. doi: 10.3389/fcimb.2019.00476 32010645 PMC6974454

[5] Yin XH Wang YD Luo H Zhao K Huang GL Luo SY. Association between tooth loss and gastric cancer: A meta-analysis of observational studies. PloS One. (2016) 11:e0149653. doi: 10.1371/journal.pone.0149653 26934048 PMC4774992

[6] Infection with helicobacter pylori. IARC Monogr Eval Carcinog Risks Hum. (1994) 61:177–240. 7715070 PMC7681529

[7] Malfertheiner P Camargo MC El-Omar E Liou JM Peek R Schulz C. Helicobacter pylori infection. Nat Rev Dis Primers. (2023) 9:1–24. doi: 10.1038/s41572-023-00431-8 37081005 PMC11558793

[8] Coker OO Dai Z Nie Y Zhao G Cao L Nakatsu G. Mucosal microbiome dysbiosis in gastric carcinogenesis. Gut. (2018) 67:1024–32. doi: 10.1136/gutjnl-2017-314281 PMC5969346 28765474

[9] Lo CH Kwon S Wang L Polychronidis G Knudsen MD Zhong R. Periodontal disease, tooth loss, and risk of oesophageal and gastric adenocarcinoma: a prospective study. Gut. (2021) 70:620–1. doi: 10.1136/gutjnl-2020-321949 PMC7855151 32690603

[10] Coussens LM Werb Z. Inflammation and cancer. Nature. (2002) 420:860–7. doi: 10.1038/nature01322 PMC2803035 12490959

[11] Sato K Takahashi N Kato T Matsuda Y Yokoji M Yamada M. Aggravation of collagen-induced arthritis by orally administered Porphyromonas gingivalis through modulation of the gut microbiota and gut immune system. Sci Rep. (2017) 7:6955. doi: 10.1038/s41598-017-07196-7 28761156 PMC5537233

[12] Walker MY Pratap S Southerland JH Farmer-Dixon CM Lakshmyya K Gangula PR. Role of oral and gut microbiome in nitric oxide-mediated colon motility. Nitric Oxide Biol Chem. (2018) 73:81–8. doi: 10.1016/j.niox.2017.06.003 PMC6104390 28602746

[13] Waghmare AS Vhanmane PB Savitha B Chawla RL Bagde HS. Bacteremia following scaling and root planing: A clinico-microbiological study. J Indian Soc Periodontol. (2013) 17:725–30. doi: 10.4103/0972-124X.124480 PMC3917200 24554880

[14] Ambrosio N Marín MJ Laguna E Herrera D Sanz M Figuero E. Detection and quantification of Porphyromonas gingivalis and Aggregatibacter actinomycetemcomitans in bacteremia induced by interdental brushing in periodontally healthy and periodontitis patients. Arch Oral Biol. (2019) 98:213–9. doi: 10.1016/j.archoralbio.2018.11.025 30503977

[15] Forner L Larsen T Kilian M Holmstrup P. Incidence of bacteremia after chewing, tooth brushing and scaling in individuals with periodontal inflammation. J Clin Periodontol. (2006) 33:401–7. doi: 10.1111/j.1600-051X.2006.00924.x 16677328

[16] O’Brien-Simpson NM Veith PD Dashper SG Reynolds EC. Porphyromonas gingivalis gingipains: the molecular teeth of a microbial vampire. Curr Protein Pept Science. (2003) 4:409–26. doi: 10.2174/1389203033487009 14683427

[17] Imamura T. The role of gingipains in the pathogenesis of periodontal disease. J Periodontology. (2003) 74:111–8. doi: 10.1902/jop.2003.74.1.111 12593605

[18] Liu Y Wu Z Nakanishi Y Ni J Hayashi Y Takayama F. Infection of microglia with Porphyromonas gingivalis promotes cell migration and an inflammatory response through the gingipain-mediated activation of protease-activated receptor-2 in mice. Sci Rep. (2017) 7:11759. doi: 10.1038/s41598-017-12173-1 28924232 PMC5603557

[19] Dominy SS Lynch C Ermini F Benedyk M Marczyk A Konradi A. Porphyromonas gingivalis in Alzheimer’s disease brains: Evidence for disease causation and treatment with small-molecule inhibitors. Sci Adv. (2019) 5:eaau3333. doi: 10.1126/sciadv.aau3333 30746447 PMC6357742

[20] Hu Y Li H Zhang J Zhang X Xia X Qiu C. Periodontitis induced by P. gingivalis-LPS is associated with neuroinflammation and learning and memory impairment in sprague-dawley rats. Front Neurosci. (2020) 14:658. doi: 10.3389/fnins.2020.00658 32714134 PMC7344110

[21] Ahn J Segers S Hayes RB. Periodontal disease, Porphyromonas gingivalis serum antibody levels and orodigestive cancer mortality. Carcinogenesis. (2012) 33:1055–8. doi: 10.1093/carcin/bgs112 PMC3334514 22367402

[22] Hanahan D Weinberg RA. The hallmarks of cancer. Cell. (2000) 100:57–70. doi: 10.1016/S0092-8674(00)81683-9 10647931

[23] Leech AO Cruz RGB Hill ADK Hopkins AM. Paradigms lost—an emerging role for over-expression of tight junction adhesion proteins in cancer pathogenesis. Ann Trans Med. (2015) 3:184–4. doi: 10.3978/j.issn.2305-5839.2015.08.01 PMC4543333 26366401

[24] Takeuchi H Amano A. Invasion of gingival epithelial cells by porphyromonas gingivalis. Methods Mol Biol. (2021) 2210:215–24. doi: 10.1007/978-1-0716-0939-2_21 32815142

[25] Bhat AA Uppada S Achkar IW Hashem S Yadav SK Shanmugakonar M. Tight junction proteins and signaling pathways in cancer and inflammation: A functional crosstalk. Front Physiol. (2018) 9:1942. doi: 10.3389/fphys.2018.01942 30728783 PMC6351700

[26] Martin TA Jiang WG. Loss of tight junction barrier function and its role in cancer metastasis. Biochim Biophys Acta (BBA) - Biomembranes. (2009) 1788:872–91. doi: 10.1016/j.bbamem.2008.11.005 19059202

[27] Rendón-Huerta EP García-García CA Estrada LFM Rendón-Huerta EP García-García CA Estrada LFM. Effect of Helicobacter pylori on Tight Junctions in Gastric Epithelia. In: Helicobacter pylori - From First Isolation to 2021 (2021) London, United Kingdom: IntechOpen. Available at: https://www.intechopen.com/chapters/75788.

[28] Martin TA Mason MD Jiang WG. Tight junctions in cancer metastasis. Front Biosci (Landmark Ed). (2011) 16:898–936. doi: 10.2741/3726 21196209

[29] Nehme Z Roehlen N Dhawan P Baumert TF. Tight junction protein signaling and cancer biology. Cells. (2023) 12:243. doi: 10.3390/cells12020243 36672179 PMC9857217

[30] Kapranos N Kominea A Konstantinopoulos P Savva S Artelaris S Vandoros G. Expression of the 27-kDa heat shock protein (HSP27) in gastric carcinomas and adjacent normal, metaplastic, and dysplastic gastric mucosa, and its prognostic significance. J Cancer Res Clin Oncol. (2002) 128:426–32. doi: 10.1007/s00432-002-0357-y PMC12164492 12200599

[31] Katsogiannou M Andrieu C Rocchi P. Heat shock protein 27 phosphorylation state is associated with cancer progression. Front Genet. (2014) 5:346. doi: 10.3389/fgene.2014.00346 25339975 PMC4186339

[32] Choi SK Kam H Kim KY Park SI Lee YS. Targeting heat shock protein 27 in cancer: A druggable target for cancer treatment? Cancers (Basel). (2019) 11:1195. doi: 10.3390/cancers11081195 31426426 PMC6721579

[33] Liu T Liu D Kong X Dong M. Clinicopathological significance of heat shock protein (HSP) 27 expression in gastric cancer: A updated meta-analysis. Evid Based Complement Alternat Med. (2020) 2020:7018562. doi: 10.1155/2020/7018562 32774426 PMC7396065

[34] Drexler R Wagner KC Küchler M Feyerabend B Kleine M Oldhafer KJ. Significance of unphosphorylated and phosphorylated heat shock protein 27 as a prognostic biomarker in pancreatic ductal adenocarcinoma. J Cancer Res Clin Oncol. (2020) 146:1125–37. doi: 10.1007/s00432-020-03175-0 PMC7142055 32200459

[35] Meghil MM Tawfik OK Elashiry M Rajendran M Arce RM Fulton DJ. Disruption of Immune Homeostasis in Human Dendritic Cells via Regulation of Autophagy and Apoptosis by Porphyromonas gingivalis. Front Immunol. (2019) 10:2286. doi: 10.3389/fimmu.2019.02286 31608069 PMC6769118

[36] Hasegawa Y Nagano K. Porphyromonas gingivalis FimA and Mfa1 fimbriae: Current insights on localization, function, biogenesis, and genotype. Jpn Dent Sci Rev. (2021) 57:190–200. doi: 10.1016/j.jdsr.2021.09.003 34691295 PMC8512630

[37] Inaba H Sugita H Kuboniwa M Iwai S Hamada M Noda T. Porphyromonas gingivalis promotes invasion of oral squamous cell carcinoma through induction of proMMP9 and its activation. Cell Microbiol. (2014) 16:131–45. doi: 10.1111/cmi.2014.16.issue-1 PMC3939075 23991831

[38] Inaba H Yoshida S Nomura R Kato Y Asai F Nakano K. Porphyromonas gulae lipopolysaccharide elicits inflammatory responses through toll-like receptor 2 and 4 in human gingivalis epithelial cells. Cell Microbiol. (2020) 22:e13254. doi: 10.1111/cmi.13254 32827217

[39] Liu XB Gao ZY Sun CT Wen H Gao B Li SB. The potential role of P.gingivalis in gastrointestinal cancer: a mini review. Infect Agents Cancer. (2019) 14:23. doi: 10.1186/s13027-019-0239-4 PMC6734237 31516546

[40] Hajishengallis G Tapping RI Harokopakis E Nishiyama S Ratti P Schifferle RE. Differential interactions of fimbriae and lipopolysaccharide from Porphyromonas gingivalis with the Toll-like receptor 2-centred pattern recognition apparatus. Cell Microbiol. (2006) 8:1557–70. doi: 10.1111/j.1462-5822.2006.00730.x 16984411

[41] Olsen I Yilmaz Ö. Possible role of Porphyromonas gingivalis in orodigestive cancers. J Oral Microbiol. (2019) 11:1563410. doi: 10.1080/20002297.2018.1563410 30671195 PMC6327928

[42] Sobocki BK Basset CA Bruhn-Olszewska B Olszewski P Szot O Kaźmierczak-Siedlecka K. Molecular mechanisms leading from periodontal disease to cancer. Int J Mol Sci. (2022) 23:970. doi: 10.3390/ijms23020970 35055157 PMC8778447

[43] Shahoumi LA Saleh MHA Meghil MM. Virulence factors of the periodontal pathogens: tools to evade the host immune response and promote carcinogenesis. Microorganisms. (2023) 11:115. doi: 10.3390/microorganisms11010115 36677408 PMC9860638

[44] Hajishengallis G Diaz PI. Porphyromonas gingivalis: immune subversion activities and role in periodontal dysbiosis. Curr Oral Health Rep. (2020) 7:12–21. doi: 10.1007/s40496-020-00249-3 33344104 PMC7747940

[45] Zhang D Chen L Li S Gu Z Yan J. Lipopolysaccharide (LPS) of Porphyromonas gingivalis induces IL-1beta, TNF-alpha and IL-6 production by THP-1 cells in a way different from that of Escherichia coli LPS. Innate Immun. (2008) 14:99–107. doi: 10.1177/1753425907088244 18713726

[46] Hajishengallis G Sojar H Genco RJ DeNardin E. Intracellular signaling and cytokine induction upon interactions of Porphyromonas gingivalis fimbriae with pattern-recognition receptors. Immunol Invest. (2004) 33:157–72. doi: 10.1081/imm-120030917 15195695

[47] Chopra A Shiheido-Watanabe Y Eberhard J. Editorial: Porphyromonas gingivalis: molecular mechanisms of invasion, immune evasion, and dysbiosis. Front Cell Infect Microbiol. (2023) 13:1289103. doi: 10.3389/fcimb.2023.1289103 37842000 PMC10570826

[48] Chow YC Yam HC Gunasekaran B Lai WY Wo WY Agarwal T. Implications of Porphyromonas gingivalis peptidyl arginine deiminase and gingipain R in human health and diseases. Front Cell Infect Microbiol. (2022) 12:987683. doi: 10.3389/fcimb.2022.987683 36250046 PMC9559808

[49] Salazar CR Sun J Li Y Francois F Corby P Perez-Perez G. Association between selected oral pathogens and gastric precancerous lesions. PloS One. (2013) 8:e51604. doi: 10.1371/journal.pone.0051604 23308100 PMC3538744

[50] Rajilic-Stojanovic M Figueiredo C Smet A Hansen R Kupcinskas J Rokkas T. Systematic review: gastric microbiota in health and disease. Aliment Pharmacol Ther. (2020) 51:582–602. doi: 10.1111/apt.15650 32056247

[51] How KY Song KP Chan KG. Porphyromonas gingivalis: An Overview of Periodontopathic Pathogen below the Gum Line. Front Microbiol. (2016) 7:53. doi: 10.3389/fmicb.2016.00053 26903954 PMC4746253

[52] Zenobia C Hajishengallis G. Porphyromonas gingivalis virulence factors involved in subversion of leukocytes and microbial dysbiosis. Virulence. (2015) 6:236–43. doi: 10.1080/21505594.2014.999567 PMC4601496 25654623

[53] Zhang C Cleveland K Schnoll-Sussman F McClure B Bigg M Thakkar P. Identification of low abundance microbiome in clinical samples using whole genome sequencing. Genome Biol. (2015) 16:265. doi: 10.1186/s13059-015-0821-z 26614063 PMC4661937

[54] Maeshima N Fernandez RC. Recognition of lipid A variants by the TLR4-MD-2 receptor complex. Front Cell Infect Microbiol. (2013) 3:3. doi: 10.3389/fcimb.2013.00003 23408095 PMC3569842

[55] Zhao S Sun M Meng H Ji H Liu Y Zhang M. TLR4 expression correlated with PD-L1 expression indicates a poor prognosis in patients with peripheral T-cell lymphomas. Cancer Manag Res. (2019) 11:4743–56. doi: 10.2147/CMAR PMC6536125 31191027

[56] Wang Y Chung YR Eitzinger S Palacio N Gregory S Bhattacharyya M. TLR4 signaling improves PD-1 blockade therapy during chronic viral infection. PloS Pathog. (2019) 15:e1007583. doi: 10.1371/journal.ppat.1007583 30726291 PMC6380600

[57] Wu X Qian S Zhang J Feng J Luo K Sun L. Lipopolysaccharide promotes metastasis via acceleration of glycolysis by the nuclear factor-κB/snail/hexokinase3 signaling axis in colorectal cancer. Cancer Metab. (2021) 9:23. doi: 10.1186/s40170-021-00260-x 33980323 PMC8117511

[58] Tamura T Ohira M Tanaka H Muguruma K Toyokawa T Kubo N. Programmed death-1 ligand-1 (PDL1) expression is associated with the prognosis of patients with stage II/III gastric cancer. Anticancer Res. (2015) 35:5369–76. doi: 10.1186/s40170-021-00260-x 26408698

[59] Tu L Guan R Yang H Zhou Y Hong W Ma L. Assessment of the expression of the immune checkpoint molecules PD-1, CTLA4, TIM-3 and LAG-3 across different cancers in relation to treatment response, tumor-infiltrating immune cells and survival. Int J Cancer. (2020) 147:423–39. doi: 10.1002/ijc.32785 31721169

[60] Sorensen SF Zhou W Dolled-Filhart M Georgsen JB Wang Z Emancipator K. PD-L1 expression and survival among patients with advanced non-small cell lung cancer treated with chemotherapy. Transl Oncol. (2016) 9:64–9. doi: 10.1016/j.tranon.2016.01.003 PMC4800057 26947883

[61] Park JH Luchini C Nottegar A Tizaoui K Koyanagi A Ogino S. Effect of CD274 (PD-L1) overexpression on survival outcomes in 10 specific cancers: a systematic review and meta-analysis. J Clin Pathol. (2023) 76:450–6. doi: 10.1136/jcp-2023-208848 37130750

[62] Costalonga M Herzberg MC. The oral microbiome and the immunobiology of periodontal disease and caries. Immunol Lett. (2014) 162:22–38. doi: 10.1016/j.imlet.2014.08.017 25447398 PMC4346134

[63] Di Stefano M Polizzi A Santonocito S Romano A Lombardi T Isola G. Impact of oral microbiome in periodontal health and periodontitis: A critical review on prevention and treatment. Int J Mol Sci. (2022) 23:5142. doi: 10.3390/ijms23095142 35563531 PMC9103139

[64] Irfan M Delgado RZR Frias-Lopez J. The oral microbiome and cancer. Front Immunol. (2020) 11:591088. doi: 10.3389/fimmu.2020.591088 33193429 PMC7645040

[65] Tonelli A Lumngwena EN Ntusi NAB. The oral microbiome in the pathophysiology of cardiovascular disease. Nat Rev Cardiol. (2023) 20:386–403. doi: 10.1038/s41569-022-00825-3 36624275

[66] Segata N Haake SK Mannon P Lemon KP Waldron L Gevers D. Composition of the adult digestive tract bacterial microbiome based on seven mouth surfaces, tonsils, throat and stool samples. Genome Biol. (2012) 13:R42. doi: 10.1186/gb-2012-13-6-r42 22698087 PMC3446314

[67] Atarashi K Suda W Luo C Kawaguchi T Motoo I Narushima S. Ectopic colonization of oral bacteria in the intestine drives TH1 cell induction and inflammation. Science. (2017) 358:359–65. doi: 10.1126/science.aan4526 PMC5682622 29051379

[68] Choi E Chang MS Byeon SJ Jin H Jung KC Kim H. Prognostic perspectives of PD-L1 combined with tumor-infiltrating lymphocytes, Epstein-Barr virus, and microsatellite instability in gastric carcinomas. Diagn Pathol. (2020) 15:69. doi: 10.1186/s13000-020-00979-z 32498695 PMC7271517

[69] Groeger S Wu F Wagenlehner F Dansranjav T Ruf S Denter F. PD-L1 up-regulation in prostate cancer cells by porphyromonas gingivalis. Front Cell Infect Microbiol. (2022) 12:935806. doi: 10.3389/fcimb.2022.935806 35846769 PMC9277116

[70] Tan Q Ma X Yang B Liu Y Xie Y Wang X. Periodontitis pathogen Porphyromonas gingivalis promotes pancreatic tumorigenesis via neutrophil elastase from tumor-associated neutrophils. Gut Microbes. (2022) 14:2073785. doi: 10.1080/19490976.2022.2073785 35549648 PMC9116393

[71] Öğrendik M. Oral bacteria in pancreatic cancer: mutagenesis of the p53 tumour suppressor gene. Int J Clin Exp Pathol. (2015) 8:11835–6. doi: 10.1080/19490976.2022.2073785 PMC4637753 26617937

[72] Yu J Ling S Hong J Zhang L Zhou W Yin L. TP53/mTORC1-mediated bidirectional regulation of PD-L1 modulates immune evasion in hepatocellular carcinoma. J Immunother Cancer. (2023) 11:e007479. doi: 10.1136/jitc-2023-007479 38030304 PMC10689408

[73] Fischbach MA. Microbiome: focus on causation and mechanism. Cell. (2018) 174:785–90. doi: 10.1016/j.cell.2018.07.038 PMC6094951 30096310

[74] Corander J Hanage WP Pensar J. Causal discovery for the microbiome. Lancet Microbe. (2022) 3:e881–7. doi: 10.1016/S2666-5247(22)00186-0 PMC9680137 36152674

[75] Zaura E Pappalardo VY Buijs MJ Volgenant CMC Brandt BW. Optimizing the quality of clinical studies on oral microbiome: A practical guide for planning, performing, and reporting. Periodontology 2000. (2021) 85:210–36. doi: 10.1111/prd.12359 PMC7756869 33226702

[76] Rosenstein ED Greenwald RA Kushner LJ Weissmann G. Hypothesis: the humoral immune response to oral bacteria provides a stimulus for the development of rheumatoid arthritis. Inflammation. (2004) 28:311–8. doi: 10.1007/s10753-004-6641-z 16245073

[77] Wegner N Wait R Sroka A Eick S Nguyen KA Lundberg K. Peptidylarginine deiminase from Porphyromonas gingivalis citrullinates human fibrinogen and α-enolase: implications for autoimmunity in rheumatoid arthritis. Arthritis Rheumatol. (2010) 62:2662–72. doi: 10.1002/art.27552 PMC2941529 20506214

[78] Everett C Li C Wilkinson JE Nguyen LH McIver LJ Ivey K. Overview of the Microbiome Among Nurses study (Micro-N) as an example of prospective characterization of the microbiome within cohort studies. Nat Protoc. (2021) 16:2724–31. doi: 10.1038/s41596-021-00519-z PMC9240631 33883746

[79] Teles FRF Alawi F Castilho RM Wang Y. Association or causation? Exploring the oral microbiome and cancer links. J Dent Res. (2020) 99:1411–24. doi: 10.1177/0022034520945242 PMC7684840 32811287

[80] Younginger BS Mayba O Reeder J Nagarkar DR Modrusan Z Albert ML. Enrichment of oral-derived bacteria in inflamed colorectal tumors and distinct associations of Fusobacterium in the mesenchymal subtype. Cell Rep Med. (2023) 4:100920. doi: 10.1016/j.xcrm.2023.100920 36706753 PMC9975273

[81] Kaji S Irino T Kusuhara M Makuuchi R Yamakawa Y Tokunaga M. Metabolomic profiling of gastric cancer tissues identified potential biomarkers for predicting peritoneal recurrence. Gastric Cancer. (2020) 23:874–83. doi: 10.1007/s10120-020-01065-5 32219586

